# Prediction of Epstein-Barr Virus Status in Gastric Cancer Biopsy Specimens Using a Deep Learning Algorithm

**DOI:** 10.1001/jamanetworkopen.2022.36408

**Published:** 2022-10-07

**Authors:** Trinh Thi Le Vuong, Boram Song, Jin T. Kwak, Kyungeun Kim

**Affiliations:** 1 School of Electrical Engineering, Korea University, Seoul, Republic of Korea; 2 Department of Pathology, Kangbuk Samsung Hospital, Sungkyunkwan University School of Medicine, Seoul, Republic of Korea

## Abstract

**Importance:**

Epstein-Barr virus (EBV)–associated gastric cancer (EBV-GC) is 1 of 4 molecular subtypes of GC and is confirmed by an expensive molecular test, EBV-encoded small RNA in situ hybridization. EBV-GC has 2 histologic characteristics, lymphoid stroma and lace-like tumor pattern, but projecting EBV-GC at biopsy is difficult even for experienced pathologists.

**Objective:**

To develop and validate a deep learning algorithm to predict EBV status from pathology images of GC biopsy.

**Design, Setting, and Participants:**

This diagnostic study developed a deep learning classifier to predict EBV-GC using image patches of tissue microarray (TMA) and whole slide images (WSIs) of GC and applied it to GC biopsy specimens from GCs diagnosed at Kangbuk Samsung Hospital between 2011 and 2020. For a quantitative evaluation and EBV-GC prediction on biopsy specimens, the area of each class and the fraction in total tissue or tumor area were calculated. Data were analyzed from March 5, 2021, to February 10, 2022.

**Main Outcomes and Measures:**

Evaluation metrics of predictive model performance were assessed on accuracy, recall, precision, F1 score, area under the receiver operating characteristic curve (AUC), and κ coefficient.

**Results:**

This study included 137 184 image patches from 16 TMAs (708 tissue cores), 24 WSIs, and 286 biopsy images of GC. The classifier was able to classify EBV-GC image patches from TMAs and WSIs with 94.70% accuracy, 0.936 recall, 0.938 precision, 0.937 F1 score, and 0.909 κ coefficient. The classifier was used for predicting and measuring the area and fraction of EBV-GC on biopsy tissue specimens. A 10% cutoff value for the predicted fraction of EBV-GC to tissue (EBV-GC/tissue area) produced the best prediction results in EBV-GC biopsy specimens and showed the highest AUC value (0.8723; 95% CI, 0.7560-0.9501). That cutoff also obtained high sensitivity (0.895) and moderate specificity (0.745) compared with experienced pathologist sensitivity (0.842) and specificity (0.854) when using the presence of lymphoid stroma and a lace-like pattern as diagnostic criteria. On prediction maps, EBV-GCs with lace-like pattern and lymphoid stroma showed the same prediction results as EBV-GC, but cases lacking these histologic features revealed heterogeneous prediction results of EBV-GC and non–EBV-GC areas.

**Conclusions and Relevance:**

This study showed the feasibility of EBV-GC prediction using a deep learning algorithm, even in biopsy samples. Use of such an image-based classifier before a confirmatory molecular test will reduce costs and tissue waste.

## Key Points

**Question:**

Can a deep learning algorithm predict Epstein-Barr virus (EBV) status of gastric cancer using pathology images?

**Findings:**

In this diagnostic study of 326 gastric cancer specimens, the proposed deep learning algorithm obtained an area under the curve of 0.87 compared with the confirmatory EBV-encoded small RNA in situ hybridization test. The algorithm also demonstrated high sensitivity and moderate specificity compared with experienced pathologists.

**Meaning:**

These findings suggest that a deep learning algorithm could predict the EBV status of gastric cancer using pathology images and could be used as a screening test before confirmatory molecular tests.

## Teaser

This diagnostic study develops and validates a deep learning algorithm to predict Epstein-Barr virus status from pathology images of gastric cancer.

## Introduction

Epstein-Barr virus (EBV), a member of the *Herpesviridae* family, is associated with lymphoid malignant neoplasms and epithelial carcinogenesis, including gastric cancer (GC).^1^ Though EBV-associated GC (EBV-GC) accounts for approximately 9% of all GCs,^2,3,4^ EBV-GC is 1 of the 4 major molecular subtypes of GC and shows clinical characteristics distinct from non–EBV-GC, such as a higher incidence in men and in the proximal location of the stomach, a lower incidence of lymph node metastasis, and a better prognosis.^2,3,4,5,6,7,8,9,10,11^ It recently has been established that programmed cell death 1 ligand 1 expression is high in EBV-GC, 1 of the target cancers for immune checkpoint inhibitor therapy, along with microsatellite instability (MSI)–high GC.^12^

EBV-GC is confirmed by EBV-encoded small RNA (EBER) in situ hybridization (ISH) testing of GC tissue.^6,13^ A confirmatory test is recommended when EBV-GC is suspected histologically on routine hematoxylin-eosin (H-E)–stained slides. However, the EBER-ISH test has a disadvantage of high cost.^3^ Two representative histologic features are indicative of EBV-GC on H-E slides: a lace- or cord-like tumor cell arrangement and a dense lymphoid stroma, which overlap with the microscopic features of a histologic subtype of GC, carcinoma with lymphoid stroma (medullary carcinoma).^12,14^ Since the EBV positivity of carcinoma with lymphoid stroma reaches 86.1%, it is straightforward to suspect EBV-GC when these 2 histologic features present on H-E slides.^6,15^ However, it is difficult to identify EBV-GC on H-E slides when there are well or moderately differentiated conventional adenocarcinomas with scant lymphoid stroma.^6,12,14,15^ In particular, predicting EBV-GC in biopsy specimens is challenging because the contained tumor size is small, and submucosal dense lymphoid stroma is often missed. The ability to prognosticate EBV-GC in biopsy specimens is advantageous since it may motivate clinicians to perform a confirmatory test for EBV-GC. Once confirmed, early-stage EBV-GC, which usually shows an undifferentiated histology, can be treated with endoscopic submucosal dissection; for instance, EBV-GC tends to present as well-circumscribed nodular lesions with low incidence of lymph node metastasis.^3,9,10,11^ Therefore, the evaluation of EBV status in biopsy specimens as well as in the surgical tissue is important in making treatment decisions.

Recently, many studies using digitized slide images and deep learning algorithms have been conducted in the field of pathology, including classification of cancers and estimation of the molecular characteristics of tumors, such as estrogen receptor, progesterone receptor, and human epidermal growth factor receptor 2 (*ERBB2*; formerly *HER2*) expression status in breast cancer, and MSI status in gastrointestinal cancers.^16,17,18,19^ For GC, a deep learning algorithm has been applied to several applications, including histologic classification^20,21,22^ and molecular prediction.^23,24,25^ As for molecular prediction, an Inception-v3 model was used to classify variation status (*CDH1*, *ERBB2*, *KRAS*, *PIK3CA*, and *TP53* variations) from H-E-stained whole slide images (WSIs).^23^ In another study,^24^ a multistep framework based on ResNet18 was used to classify image patches from H-E–stained WSIs into benign, tumor, and EBV− and EBV+ tumor. A deep learning model was trained on tissue patches from tissue microarrays (TMAs) and WSIs to classify EBV, MSI, and other types, and a mean of randomly selected patches was used for a patient-level prediction.^25^ However, these previous studies were conducted using WSIs and/or TMAs of gastric resection specimens or using virtual biopsy techniques, which simulate tissue images of endoscopic biopsy with a 2-mm annotation, including GC and adjacent gastric mucosa areas in WSIs of resected GC specimens.^23,25,26^ There has been no prior study for predicting EBV status in GC biopsy tissues. Therefore, in this study, we sought to develop a deep learning classifier that can predict EBV-GC in biopsy specimens. The deep learning classifier was trained using GC TMAs and WSIs and was validated using GC biopsy tissues.

## Methods

This study was approved by the regional institutional review board of Kangbuk Samsung Hospital and was performed according to the ethical standards of the Declaration of Helsinki, as revised in 2008.^27^ The review conducted by our institutional review board confirmed that informed consent was not necessary because this was a retrospective study using fully anonymized data. This study followed the reporting guideline of the Transparent Reporting of a Multivariable Prediction Model for Individual Prognosis or Diagnosis (TRIPOD) reporting guideline.

### GC Specimens and Histologic Review

This study includes TMAs and WSIs obtained at Kangbuk Samsung Hospital. We initially enrolled 734 gastrectomy specimens to generate TMA blocks from tissue resected for GC between January 2011 and December 2020. One representative tumor core with a 2-mm diameter was obtained from each tumor, generating 16 TMA blocks. From this cohort, 24 WSIs were generated. We also enrolled an independent cohort with 286 biopsy specimens that were diagnosed as gastric adenocarcinoma between January 2016 and December 2020. During microscopic review, histologic features of tumor differentiation, a lace-like growth pattern, lymphoid stroma, presence of signet ring cell components, and mucin production were independently assessed by 2 experienced pathologists (K.K. and B.S.) and were determined on consensus. All gastrectomy and biopsy specimens were fixed in 10% neutral buffered formalin solution and embedded in paraffin.

### EBV in Situ Hybridization

To confirm EBV-GC, EBER-ISH was performed with all TMA blocks and biopsy cases using a BOND ready-to-use ISH EBER probe and BOND III autostainer (Leica Biosystems Nussloch) according to the manufacturer’s protocol. Cases with diffuse strong nuclear positivity were considered EBV-GC cases (eFigure 1 in the Supplement). All glass slides were scanned using a digital slide scanner (AT2, Aperio, Leica Biosystems Imaging) at 40× magnification.

### Data Set and Annotation for EBV-GC

Both TMAs and WSIs were annotated by experienced pathologists (K.K. and B.S.), who localized the regions of interest and assigned class labels, including EBV-GC, non–EBV-GC, and benign gastric tissue. The GC areas, including GC cells and intervening stroma, were classified into EBV-GC or non–EBV-GC according to the test result of EBER-ISH rather than histologic features. For annotating cancer areas, the pathologists tried to minimize the inclusion of stroma. Each TMA core had a size of approximately 8500 × 8500 pixels (0.2518 μm × 0.2518 μm per pixel), and each WSI had a size up to 100 000 × 50 000 pixels (0.2465 μm × 0.2465 μm per pixel).

Following previous works,^28,29^ we generated image patches from TMAs and WSIs via a sliding window scheme. In total, 137 184 image patches of 1024 × 1024 pixels were generated with a stride of 512 pixels for both training and testing procedures. In this manner, we sought to reduce the number of redundant image patches and focus on the patterns of tissues near the center of the patches; otherwise, some patterns only appear near the boundary. Patches with fewer than 80% of pixels belonging to a prominent class and more than 20% luminal pixels were excluded. The remaining patches were used for classification into EBV-GC, non–EBV-GC, and benign gastric tissue (eFigure 1 in the Supplement). The image patches were divided into training, validation, and test data sets, the details of which are shown in eTable 1 in the Supplement. The size of the biopsy images was up to 130 000 × 40 000 pixels (0.2521 μm × 0.2521 μm per pixel). For biopsy images, the EBV status was provided according to EBER-ISH results without region of interest marking for EBV-GC areas.

### Deep Learning Classification Model Development

For classification of EBV-GC, non–EBV-GC, and benign classes, we considered 3 convolutional neural network (CNN)–based models: ResNet,^30^ MobileNet,^31^ and EfficientNet^32^ and a vision transformer–based model, DeiT^33^ (eAppendix 1 in the Supplement). Among the 4 models, we first chose the best classification model. Then, we optimized the chosen classification model and applied it to biopsy tissue images for EBV prediction.

To select the best classification model, we conducted a 5-fold cross-validation experiment for each of the 4 classification models on the image patches obtained from TMAs and WSIs. The entire image patches were randomly divided into 5 disjoint sets. One set was used as the test set, 3 sets were used as a training set, and the last set was used as a validation set. Using the training, validation, and test sets, we chose the optimal model and assessed its performance on the test set. This process was repeated 5 times with a different choice of a test set. The performance of each model on the test sets was summarized and used to choose the best classification model.

Given the best classification model, we optimized and applied it to biopsy images for EBV prediction. The entire image patches from TMAs and WSIs were split into training, validation, and test data sets (eTable 1 in the Supplement) and used to optimize the best classification model. The optimized model was used for EBV prediction in biopsy tissue images.

### Deep Learning Classification Model Training and Evaluation

To reduce memory burden during training and inference phases, the image patches were resized to 512 × 512 pixels for CNN-based models and 384 × 384 pixels for DeiT and fed into the classification models. In the training phase, we adopted several data augmentation techniques, including a random flip, rotation, color change, and blurring, to increase the diversity of the training data set (eAppendix 2 in the Supplement). We optimized all the classification models with the Adam optimizer using default hyperparameters (β_1_ = 0.9; β_2_ = 0.9; ε = 1.0 × 10^−8^), a batch size of 64, a cross-entropy objective function, and a cosine annealing warm restarts scheduler^34^ using the initial learning rate to 1.0 × 10^−3^ (eAppendix 2 in the Supplement). The CNN-based classification models were trained for 60 epochs, and DeiT was trained for 120 epochs. All the classification models were implemented using the Pytorch library and then trained on a workstation with 4 RTX 3090 GPUs (NVIDIA).

On completion of model training, we applied the trained model to the image patches, which were resized as described, in the validation data set and chose the optimal classification model that achieved the best performance. Then, the optimal model was used to assess the classification performance on the test data set.

### Performance Evaluation

We used 5 evaluation metrics to quantitatively assess the performance of the classification model at the patch level: accuracy, mean recall, mean precision, macromean F1 score, and κ coefficient. Moreover, we visualized the classification results on TMAs, WSIs, and biopsy tissue images as prediction maps by adopting a sliding window strategy. The size of the window was set to 1024 × 1024 pixels with a stride of 256 pixels for WSIs and biopsy tissue images and 128 pixels for TMAs. The classification model computed the probability of benign, EBV-GC, and non–EBV-GC classes and the highest probability among these classes was assigned as the predicted class on a per-pixel basis in the prediction map. In addition, we computed the areas of the EBV-GC, non–EBV-GC, and benign classes in each prediction map and calculated the fraction of EBV-GC over the entire tissue area or the entire tumor area eAppendix 3 in the Supplement).

### Statistical Analysis

Statistical significance in discriminating between EBV-GC and non–EBV-GC samples was determined by the Wilcoxon rank-sum test. Pearson correlations were adopted to measure the correlations between EBV status and histological features. Bootstrap resampling with 2000 repetitions was used to compute the 95% CIs for the areas under the receiver operating characteristic curves (AUCs) and the statistical significance between 2 AUCs. Differences were considered statistically significant at 2-sided *P* < .05. Data were analyzed using R software version 4.12 (R Project for Statistical Computing). Data were analyzed from March 5, 2021, to February 10, 2022.

## Results

### Results of EBV-GC Prediction by Deep Learning Model for Patch Images

This study includes 137 184 image patches from 16 TMAs (708 tissue cores), 24 WSIs, and 286 biopsy images of GC. Table 1 shows the patch-level classification results of the 4 models (ResNet,^30^ MobileNet,^31^ EfficientNet,^32^ and DeiT^33^). In a 5-fold cross-validation experiment, EfficientNet was superior to other models, with an mean accuracy of 93.70%, recall of 0.923, precision of 0.925, F1 score of 0.921, and κ coefficient of 0.896, and was chosen as the best classification model. Then, EfficientNet was optimized, achieving 94.70% for accuracy, 0.936 for recall, 0.938 for precision, 0.937 for F1 score, and 0.909 for κ coefficient on the test dataset from TMAs and WSIs, and used for EBV-GC prediction in biopsy images. The confusion matrix clearly showed that the selected deep learning model (ie, EfficientNet) performed well and was not biased toward a particular class (eTable 2 in the Supplement). Representative prediction maps for TMAs and WSIs are presented in eFigure 2 in the Supplement.

**Table 1.** Results of Patch-Level EBV-GC Prediction

Measure	Accuracy, %	Recall	Precision	F1	κ Score
**5-Fold cross validation, mean (SD)**					
MobileNet	93.60 (1.20)	0.923 (0.021)	0.925 (0.007)	0.923 (0.014)	0.893 (0.020)
ResNet	91.40 (2.40)	0.896 (0.035)	0.904 (0.020)	0.897 (0.029)	0.858 (0.038)
DeiT	92.50 (0.90)	0.913 (0.016)	0.912 (0.014)	0.912 (0.012)	0.875 (0.018)
EfficientNet	93.70 (1.80)	0.923 (0.030)	0.925 (0.021)	0.921 (0.029)	0.896 (0.030)
**Testing data set, estimate**					
MobileNet	90.70	0.892	0.891	0.892	0.842
ResNet	92.00	0.890	0.914	0.900	0.862
DeiT	93.50	0.916	0.931	0.923	0.889
EfficientNet	94.70	0.936	0.938	0.937	0.909

Abbreviation: EBV-GC, EBV-associated gastric cancer.

### Results of EBV-GC Prediction by Deep Learning Model for Biopsy Images

Based on the EBER-ISH test results, 19 specimens (6.6%) were diagnosed as EBV-GC and 267 specimens (93.4%) were diagnosed as non–EBV-GC. In a head-to-head comparison of histological findings between EBV-GC and non–EBV-GC, 2 well-known histologic features for EBV-GC, including lace-like pattern and lymphoid stroma, were highly correlated to EBV-GC specimens, and the presence of signet ring cell components was correlated with non–EBV-GC specimens (eTable 3 in the Supplement). However, cases showing both a lace-like pattern and a lymphoid stroma represented less than half of the specimens (9 of 19 specimens). Lymphoid stroma was observed in only 57.9% of EBV-GC cases. At the time of biopsy diagnosis, approximately two-thirds of the EBV-GC specimens were diagnosed as tubular adenocarcinoma with poor differentiation, without mention of the possibility of EBV-GC.

To visually assess the prediction results, we investigated the prediction maps for biopsy images. Among EBV-GC specimens, the biopsy samples with lace-like pattern and lymphoid stroma showed homogeneous prediction results compared with EBV-GC on the prediction map (eFigure 3 in the Supplement). Some cases lacking a lace-like pattern or lymphoid stroma revealed heterogeneous prediction results admixed with EBV-GC and non–EBV-GC on the prediction maps, although there was no distinct histologic difference between prediction areas classified as EBV-GC and non–EBV-GC even at high magnification (eFigure 3 in the Supplement). Interestingly, a non–EBV-GC biopsy case with marked neutrophilic infiltration was well predicted as non–EBV-GC despite dense intraepithelial and stromal infiltration (eFigure 3 in the Supplement).

Moreover, we calculated a number of measurements, including the areas of EBV-GC, non–EBV-GC, total tumor, and benign tissues; the fractions of EBV-GC, non–EBV-GC, and benign tissues in the total tissue area; and the fraction of EBV-GC area in the total tumor area (Table 2). We calculated the means and compared between the ground truth EBV-GC and non–EBV-GC. The predicted EBV-GC area was higher than in the ground truth EBV-GC (213 992 μm^2^ vs 511 466 μm^2^; *P* < .001), but there were no significant differences in areas of non–EBV-GC, tumor, and benign gastric tissue. In the fraction measurements, the predicted EBV-GC/tissue (31.7%) and EBV-GC/tumor (58.0%) were higher than in the ground truth EBV-GC (EBV-GC/tissue: 7.3%; EBV-GC/tumor: 19.6%; *P* < .001), and the predicted non–EBV-GC/tissue and benign/tissue were higher than in the ground truth non–EBV-GC. In the AUC analysis of these measurements, the predicted area of EBV-GC and the predicted fraction of EBV-GC to tissue and tumor showed high AUC values compared with the remaining measurements (Table 3; eFigure 4 in the Supplement).

**Table 2.** Summary of EBV-GC Prediction on an Independent Biopsy Tissue Data Set

Prediction results	Ground truth, mean (SD)		*P* value
	EBV-GC	Non–EBV-GC	
**Area, μm^2^**			
EBV-GC	213 992 (191 189)	51 466 (83 384)	<.001
Non–EBV-GC	128 712 (114 704)	205 199 (205 177)	.08
Tumor^a^	342 704 (257 846)	256 665 (229 546)	.11
Benign	315 467 (187 018)	435 340 (291 268)	.13
**Fraction, %**			
EBV-GC/tissue	31.7 (18.5)	7.3 (10.4)	<.001
Non–EBV-GC/tissue	20.1 (16.3)	30.9 (22.0)	.04
EBV-GC/tumor^a^	58.0 (27.9)	19.6 (23.9)	<.001
Benign/tissue	48.5 (21.4)	61.8 (23.4)	.008

Abbreviations: EBV, Epstein-Barr virus; EBV-GC, EBV-associated gastric cancer.

^a^ Tumor includes both EBV-GC and non–EBV-GC.

**Table 3.** AUC Analysis for EBV-GC Prediction on a Biopsy Tissue Data Set

Prediction results	AUC (95% CI)	*P* value		
		EBV-GC area, μm^2^	EBV-GC/tissue, %	EBV-GC/tumor, %
**Area, μm^2^**				
EBV-GC	0.8257 (0.7183-0.9154)	NA	.08	.59
Non–EBV-GC	0.6185 (0.4854-0.7556)	.04	.005	.01
Tumor^a^	0.6103 (0.4652-0.7428)	<.001	<.001	.001
Benign	0.6040 (0.4908-0.7207)	.01	<.001	.003
**Fraction**				
EBV-GC/tissue	0.8723 (0.7560-0.9501)	.08	NA	.059
Non–EBV-GC/tissue	0.6413 (0.5222-0.7550)	.02	.004	.006
EBV-GC/tumor^a^	0.8396 (0.7289-0.9210)	.58	.07	NA
Benign/tissue	0.6813 (0.5623-0.7885)	.008	<.001	.005

Abbreviations: AUC, area under the receiver operating characteristic curve; EBV, Epstein-Barr virus; EBV-GC, EBV-associated gastric cancer; NA not applicable.

^a^ Tumor includes both EBV-GC and non–EBV-GC.

To compare the performance of our deep learning model with that of the known pathologic features for EBV-GC in identifying EBV-GC specimens, we calculated sensitivity and specificity for the pathologic features and the prediction results with differing threshold values (Table 4). For the pathologic features, suspicion of EBV-GC in the presence of a lace-like pattern or lymphoid stroma had the highest sensitivity (0.842) and specificity (0.854). For the deep learning prediction results, both sensitivity and specificity were considered to determine the optimal cutoff value. On one hand, the cutoff value needs to achieve high sensitivity to accurately identify EBV-GC specimens, and on the other hand the cutoff value needs to obtain high specificity to reduce tissue waste and medical cost. Since EBV-GC is a rare subtype accounting for less than 10% of GC, and EBER-ISH, a confirmatory test, is an expensive molecular test, the cutoff value with low specificity will result in a substantial number of false positives, leading to increase in unnecessary EBER-ISH tests; for instance, 0.506 specificity by EBV-GC/tumor indicates that approximately 45% of the entire GC population will be subject to EBER-ISH but none of them will have EBV-GC. The optimal cutoff values, achieving the highest mean sensitivity and specificity, were 5000 μm^2^ for EBV-GC, 10% of EBV-GC/tissue, and 30% of EBV-GC/tumor, and the best performance was obtained using 10% of EBV-GC/tissue with high sensitivity (0.895) and moderate specificity (0.745).

**Table 4.** Comparison of EBV-GC Prediction and Case Distribution on a Biopsy Tissue Data Set

Measure	Threshold	Sensitivity	Specificity	(Sensitivity + specificity) / 2	Distribution of cases			
					EBV-GC (n = 19)		Non–EBV-GC (n = 267)	
					True positive	False negative	True negative	False positive
Microscopic features by pathologists								
Lace-like pattern alone	NA	0.789	0.888	0.839	15	4	237	30
Lymphoid stroma alone	NA	0.579	0.933	0.756	11	8	249	18
Lace-like pattern or lymphoid stroma	NA	0.842	0.854	0.848	16	3	228	39
Lace-like pattern and lymphoid stroma	NA	0.474	0.966	0.720	9	10	258	9
Prediction by deep learning model								
EBV-GC, μm^2^	10 000	0.947	0.416	0.682	18	1	111	156
	50 000	0.842	0.693	0.768	16	3	185	82
	90 000	0.684	0.813	0.749	13	6	217	50
EBV-GC/tissue, %	10%	0.895	0.745	0.820	17	2	199	68
	20%	0.684	0.891	0.788	13	6	238	29
	30%	0.579	0.948	0.764	11	8	253	14
EBV-GC/tumor, %^a^	10%	0.947	0.506	0.727	18	1	135	132
	20%	0.895	0.655	0.775	17	2	175	92
	30%	0.842	0.753	0.798	16	3	201	66

Abbreviations: EBV, Epstein-Barr virus; EBV-GC, EBV-associated gastric cancer; NA, not applicable.

^a^ Tumor includes both EBV-GC and non–EBV-GC.

## Discussion

This diagnostic study is the first to evaluate the feasibility of EBV-GC prediction in GC biopsy specimens, to our knowledge. Our deep learning algorithm showed high sensitivity and specificity even in small GC biopsy specimens, supporting the feasibility and validity of our study. The results suggest that this can facilitate automated detection of EBV-GC in clinics, supporting decisions of GC specialists and assisting GC nonspecialists in suspecting and identifying EBV-GC, which could help to improve treatment planning and decision-making.

Suspicion of EBV-GC in the diagnosis of GC in biopsy specimens and induction of confirmatory tests have several added values in clinics. First, EBV-GC has a lower lymph node metastasis rate compared with non–EBV-GC. The identification of EBV status before surgery would be useful to determine the optimal cancer removal method, especially for early-stage GC, because an endoscopic resection could be considered despite undifferentiated histology at biopsy diagnosis. Second, for patients with advanced GC who receive neoadjuvant chemotherapy or only chemotherapy, various ancillary molecular tests must be performed with only small quantities of biopsy tissues. The ability to predict EBV status from a biopsy specimen would be advantageous in preventing tissue waste. Third, using a deep learning algorithm as a screening test before a confirmatory test would contribute to cost reduction in pathology service.

Diagnosing or subtyping GC in biopsy specimens is a harder task for pathologists than diagnosing it in resection specimens for several reasons; for example, the amount of tissue is small, and accompanying surface changes, such as ulcers or erosions and squeezing artifacts due to biopsy forceps, could alter the structural and cellular morphology.^35,36^ A previous study for EBV-GC prediction in whole tissue areas, tumor-only areas, and virtual biopsy areas of luminal slides revealed that the AUC decreased significantly in the virtual biopsy areas compared with whole-slide areas.^26^ Similarly, our deep learning algorithm showed better performance for EBV-GC in the patch-level prediction using TMAs and WSIs (94.7% accuracy) but demonstrated similar or lower performance on biopsy tissue images than pathologist estimation. This suggests that the histologic differences between the tumor central area and the luminal surface could affect the performance of a deep learning algorithm. Since we trained the algorithm on resected GC tissues, adding more biopsy cases will improve the overall performance of EBV prediction.

In histology, prominent lymphoid stroma and lace pattern components are major characteristics of EBV-GC and were observed in 84.5% of resected EBV-GC cases in a previous report.^14^ Specifically, for lymphoid infiltration, even the spatial pattern on the lymphoid infiltration map contributed to determination of EBV infection status.^25^ In our study, cases showing both a lace-like pattern and a lymphoid stroma represented less than 50% (9 of 19). Lymphoid stroma was observed in only 57.9% of EBV-GC cases, and the density of lymphoid infiltration was low without lymphoid follicles. The lace-like pattern was observed only in a small portion of GC and is likely to be diagnosed as poorly differentiated adenocarcinoma at biopsy. The 2 histologic features were important for predicting EBV-GC at biopsy, although they were infrequent in biopsy specimens. Furthermore, some cases with conventional histologic features of medullary carcinoma were correctly predicted as EBV-GC with a homogeneous prediction map, but other cases lacking lymphoid stroma or showing conventional adenocarcinoma histology showed heterogeneous results in the prediction maps, suggesting the influence of these 2 histologic features on our deep learning classifier. Interestingly, a non–EBV-GC case with marked neutrophil infiltration was well classified as non–EBV-GC. This suggests that our classifier is capable of recognizing infiltrating cells among tumor cells, leading to further subtyping of GC.

### Limitations

There are several limitations in this study. First, deep learning prediction results were not associated with histologic features, such as lymphoid stroma and lace-like patterns, in particular for heterogeneous results in the prediction maps, due to the absence of quantitative measurement and regional annotations for them. Digital pathology analysis, including texture and morphometry, may be applied to discover new features associated with EBV-GC. However, the new features need to be either associated with known histologic features or clinically confirmed by an external validation study to allow an objective and concrete conclusion. Second, this study included only GC cases from a single institute in Korea. To further validate our approach, a large-scale follow-up study is desirable. Third, 4 existing deep learning algorithms^30,31,32,33^ were considered for EBV prediction. We may be able to improve classification performance by adopting other architectures or designing a specialized architecture for EBV-GC. Fourth, the deep learning classifier was developed using patch-level ground truth labels. Using pixel-level annotations, the classifier might be able to provide finer prediction maps, leading to improved decision making.

## Conclusions

In this diagnostic study, we developed a deep learning classifier that predicted EBV status in H-E–stained slides, demonstrating good performance with GC biopsy specimens. This classifier could help pathologists to suspect EBV-GC in biopsy diagnoses, leading to confirmation using EBER-ISH. Additionally, such an attempt to predict subtypes at biopsy specimen level would help clinicians both in establishing a treatment strategy before surgery or endoscopic resection and in selecting chemotherapy regimens.

## Notes

Supplement. **eAppendix 1.** Deep Learning Classification Model **eAppendix 2.** Deep Learning Classification Model Training and Evaluation **eAppendix 3.** Performance Evaluation **eTable 1.** Detailed Number of Images for Classification Model Development **eTable 2.** Confusion Matrix for EBV Prediction on the Patch-Level Testing Data Set **eTable 3.** Histologic Features of 286 Adenocarcinoma Biopsy Specimens According to EBV Status **eFigure 1.** Representative Images for Patch Extraction of EBV-GC, Non–EBV-GC, and Benign Gastric Tissue **eFigure 2.** Representative Prediction Results With TMAs and WSIs **eFigure 3.** Representative Prediction Maps of Biopsy Specimens **eFigure 4.** ROC Curves for EBV-GC Prediction in Biopsy Specimens
eReferences.
