# Supplementary material for: Prediction of Epstein-Barr Virus Status in Gastric Cancer Biopsy Specimens Using a Deep Learning Algorithm
Source: JAMA Netw Open. 2022 Oct 7;5(10):e2236408. doi: 10.1001/jamanetworkopen.2022.36408 (PMC9547324; doi:10.1001/jamanetworkopen.2022.36408)
Supplement: Supplement. — eAppendix 1. Deep Learning Classification Model eAppendix 2. Deep Learning Classification Model Training and Evaluation eAppendix 3. Performance Evaluation eTable 1. Detailed Number of Images for Classification Model Development eTable 2. Confusion Matrix for EBV Prediction on the Patch-Level Testing Data Set eTable 3. Histologic Features of 286 Adenocarcinoma Biopsy Specimens According to EBV Status eFigure 1. Representative Images for Patch Extraction of EBV-GC, Non–EBV-GC, and Benign Gastric Tissue eFigure 2. Representative Prediction Results With TMAs and WSIs eFigure 3. Representative Prediction Maps of Biopsy Specimens eFigure 4. ROC Curves for EBV-GC Prediction in Biopsy Specimens eReferences. [file jamanetwopen-e2236408-s001.pdf]

Supplemental Online Content

Vuong TTL, Song B, Kwak JT, Kim K. Prediction of Epstein-Barr virus status in gastric cancer biopsy specimens using a deep learning algorithm. *JAMA Netw Open*. 2022;5(10):e2236408. doi:10.1001/jamanetworkopen.2022.36408

eAppendix 1. Deep Learning Classification Model

eAppendix 2. Deep Learning Classification Model Training and Evaluation

eAppendix 3. Performance Evaluation

eTable 1. Detailed Number of Images for Classification Model Development

eTable 2. Confusion Matrix for EBV Prediction on the Patch-Level Testing Data Set

eTable 3. Histologic Features of 286 Adenocarcinoma Biopsy Specimens According to EBV Status

eFigure 1. Representative Images for Patch Extraction of EBV-GC, Non–EBV-GC, and Benign Gastric Tissue

eFigure 2. Representative Prediction Results With TMAs and WSIs

eFigure 3. Representative Prediction Maps of Biopsy Specimens

eFigure 4. ROC Curves for EBV-GC Prediction in Biopsy Specimens

eReferences.

This supplemental material has been provided by the authors to give readers additional information about their work.

© 2022 Vuong TTL et al. *JAMA Network Open.*

eAppendix 1. Deep Learning Classification Model

For classification of EBV-GC, non-EBV-GC, and benign classes, we considered the three CNN-based models ResNet,[1] MobileNet,[2] EfficientNet[3] and a vision transformer (ViT)-based model, DeiT.[4] ResNet[1] is built based upon a skip connection that has been wildly adopted for many computer vision-related tasks. Among its variants, we employed ResNet50, which utilizes a set of 1 x 1, 3 x 3, and 1 x 1 convolutional layers that are repeated 3, 4, 6, and 3 times. MobileNet[2] is a lightweight network that is based upon the mobile inverted bottleneck convolution (MBconv) block and is suitable for small (medical) image datasets. We utilized MobileNetV2 in this study. The MBconv block employed a narrow-wide-narrow approach using an expansion 1 × 1 convolution, a depthwise convolution layer, a squeeze and excitation (SE) layer,[5] and a compression 1 × 1 convolution layer. The output dimension of the compression layer matched the input dimension. In the MBConv block, the convolutional layer was followed by a batch normalization layer and a swish activation function.[6] EfficientNet refers to a group of neural networks that utilize the compound scaling method to increase the width, depth, and resolution of the network based on a fixed scaling factor. The networks are primarily based upon MBconv blocks. Among several variants, we used EfficientNetB1 in this study. EfficientNetB1 includes one 3 × 3 convolutional layer, one MBconv1 block, 15 MBconv6 blocks, one 1 × 1 convolutional layer, one pooling layer, and one fully connected layer. The MBconv1 block and MBconv6 blocks denote MBconv blocks with expansion factors of 1 and 6, respectively. DeiT[4] refers to data-efficient image transformers based upon a vision transformer (ViT).[7] DeiT decomposes an input image into a set of image tiles of size 16 x 16 pixels, projects each image tile with a linear layer, and adds positional information, generating input tokens. These tokens are fed into a stack of transformer blocks, each of which consists of two linear layers and multi-head self-attention layers. For image classification, a class token is appended to the input tokens and is processed by the transformer blocks and linear layer to produce a prediction.

© 2022 Vuong TTL et al. *JAMA Network Open.*

eAppendix 2. Deep Learning Classification Model Training and Evaluation

To reduce memory burden during training and inference phases, the image patches were resized to 512 × 512 pixels for CNN-based models and 384 x 384 pixels for DeiT and fed into the classification models. In the training phase, we adopted several data augmentation techniques: 1) a random flip along a horizontal or vertical direction; 2) a random affine transformation with a shear in the range [-16º, 16º] or a rotation in the range [-45º, 45º]; 3) changes in hue, saturation, and contrast of images; 4) random blurring with a Gaussian, average, or median filter; and 5) random additive noise and lost pixels. The aleju library (https://github.com/aleju/imgaug) was employed to conduct these augmentations.

During training, we optimized all the classification models with the Adam optimizer using default hyperparameters (β_1=0.9, β_2=0.9, ε=1.0E^{-8}), a batch size of 64, and a cross-entropy objective function. The CNN-based classification models were trained for 60 epochs, and DeiT was trained for 120 epochs. For all the classification models, a cosine annealing warm restarts scheduler[8] was utilized by setting the initial learning rate to 1.0e^{-3} and the number of iterations for the first restart T_0 to 20. The weights of the classification models were initialized using the pre-trained weights on the ImageNet dataset except for those in the last layers, which were initialized using the K. He method.[9] All the classification models were implemented using the Pytorch library and then trained on a workstation with four RTX 3090 GPUs.

Upon completion of model training, we applied the trained model to the image patches, which were resized as above, in the validation dataset and chose the optimal classification model that achieved the best performance. Then, the optimal model was utilized to assess the classification performance on the test dataset.

© 2022 Vuong TTL et al. *JAMA Network Open.*

eAppendix 3. Performance Evaluation

We utilized five evaluation metrics to quantitatively assess the performance of the classification model at the patch level: 1) accuracy (Acc): overall classification accuracy among all classes; 2) average recall (Recall): arithmetic mean of per-class recall; 3) average precision (Precision): arithmetic mean of per-class precision; 4) macro-averaged F1 (F1): arithmetic mean of per-class recall, precision, and F1-scores; and 5) kappa coefficient (Kappa): $\kappa = \frac{p_o - p_e}{1 - p_e}$, where p_o is the overall accuracy, $p_e = \frac{1}{N^2}\sum_c n_{ct} n_{cp}$, $c = 3$ (the number of classes), n_{ct} is the number of samples of class c, and n_{cp} is the number of samples predicted as class c.

Moreover, we visualized the classification results on TMAs, WSIs, and biopsy tissue images as prediction maps by adopting a sliding window strategy. For each tissue image, a rectangular window moved from the left-top corner to the right-bottom corner of the image with a stride of 256 pixels for WSIs and biopsy tissue images and 128 pixels for TMAs. The size of the window was set to 1024 × 1024 pixels, generating an image patch that was resized by half and fed into the classification model. The classification model computed the probability of benign, EBV-GC, and non-EBV-GC classes for each rectangular window. If multiple rectangular windows passed through a region, the probabilities were averaged among the overlapping windows. Here, we obtained three probability maps for each of the tissue images that corresponded to the benign, EBV-GC, and non-EBV-GC classes. Then, the highest probability among these classes was assigned as the predicted class on a per-pixel basis in the final prediction map. In addition, we computed the areas of the EBV-GC, non-EBV-GC, and benign classes in each prediction map and calculated the fraction of EBV-GC over the entire tissue area or the entire tumor area.

© 2022 Vuong TTL et al. *JAMA Network Open.*

eTable 1. Detailed Number of Images for Classification Model Development

	TMA	WSI	Patch images			
			EBV-GC	Non-EBV-GC	Benign	Total
Training	376	14	17285	19406	35523	72214
Validation	168	5	7004	7749	16413	31166
Testing	164	5	6640	8156	19008	33804

Abbreviations: TMA, tissue microarray; WSI, whole-slide image; EBV-GC, EBV-associated gastric cancer; Non-EBV-GC, non-EBV-associated gastric cancer.

© 2022 Vuong TTL et al. *JAMA Network Open.*

eTable 2. Confusion Matrix for EBV Prediction on the Patch-Level Testing Data Set

	Ground Truth		
	EBV-GC (%)	Non-EBV-GC (%)	Benign (%)
Prediction			
EBV-GC	90.80	2.20	1.30
Non-EBV-GC	4.70	93.10	1.90
Benign	4.50	4.70	96.70

Abbreviations: EBV-GC, EBV-associated gastric cancer; Non-EBV-GC, non-EBV-associated gastric cancer.

© 2022 Vuong TTL et al. *JAMA Network Open.*

eTable 3. Histologic Features of 286 Adenocarcinoma Biopsy Specimens According to EBV Status

	EBV-GC	Non-EBV-GC	*p*-value
Histologic diagnosis			0.800
Tubular, well differentiated	1 (5.30%)	54 (20.2%)	
Tubular, moderately differentiated	6 (31.60%)	83 (31.10%)	
Tubular, poorly differentiated	12 (63.20%)	63 (23.60%)	
Poorly cohesive	0 (0.00%)	67 (25.10%)	
Lace-like pattern			<0.001
Absent	4 (21.10%)	237 (88.80%)	
Present	15 (78.90%)	30 (11.20%)	
Lymphoid stroma			<0.001
Absent	8 (42.10%)	249 (93.30%)	
Present	11 (57.90%)	18 (6.7%)	
Signet ring cell component			0.012
Absent	18 (94.70%)	180 (67.4%)	
Present, less than 50%	1 (5.30%)	48 (20.0%)	
Present, more than 50%	0 (0.00%)	39 (14.6%)	
Mucin component			0.348
Absent	19 (100.00%)	255 (95.5%)	
Present	0 (0.00%)	12 (4.5%)	

Abbreviations: EBV-GC, EBV-associated gastric cancer; Non-EBV-GC, non-EBV-associated gastric cancer.

© 2022 Vuong TTL et al. *JAMA Network Open.*

eFigure 1. Representative Images for Patch Extraction of EBV-GC, Non–EBV-GC, and Benign Gastric Tissue

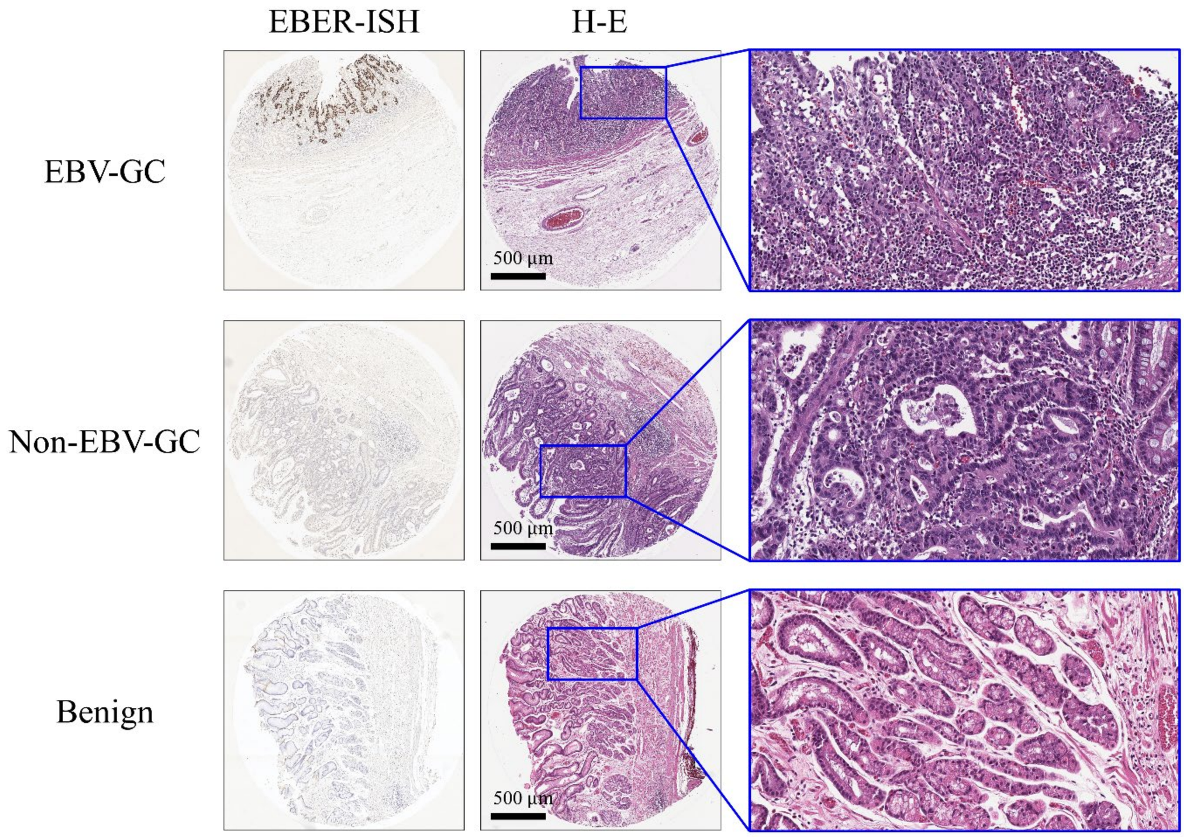

EBV status is checked through EBER-ISH, and patch images are extracted for each class from the corresponding area.

© 2022 Vuong TTL et al. *JAMA Network Open.*

eFigure 2. Representative Prediction Results With TMAs and WSIs

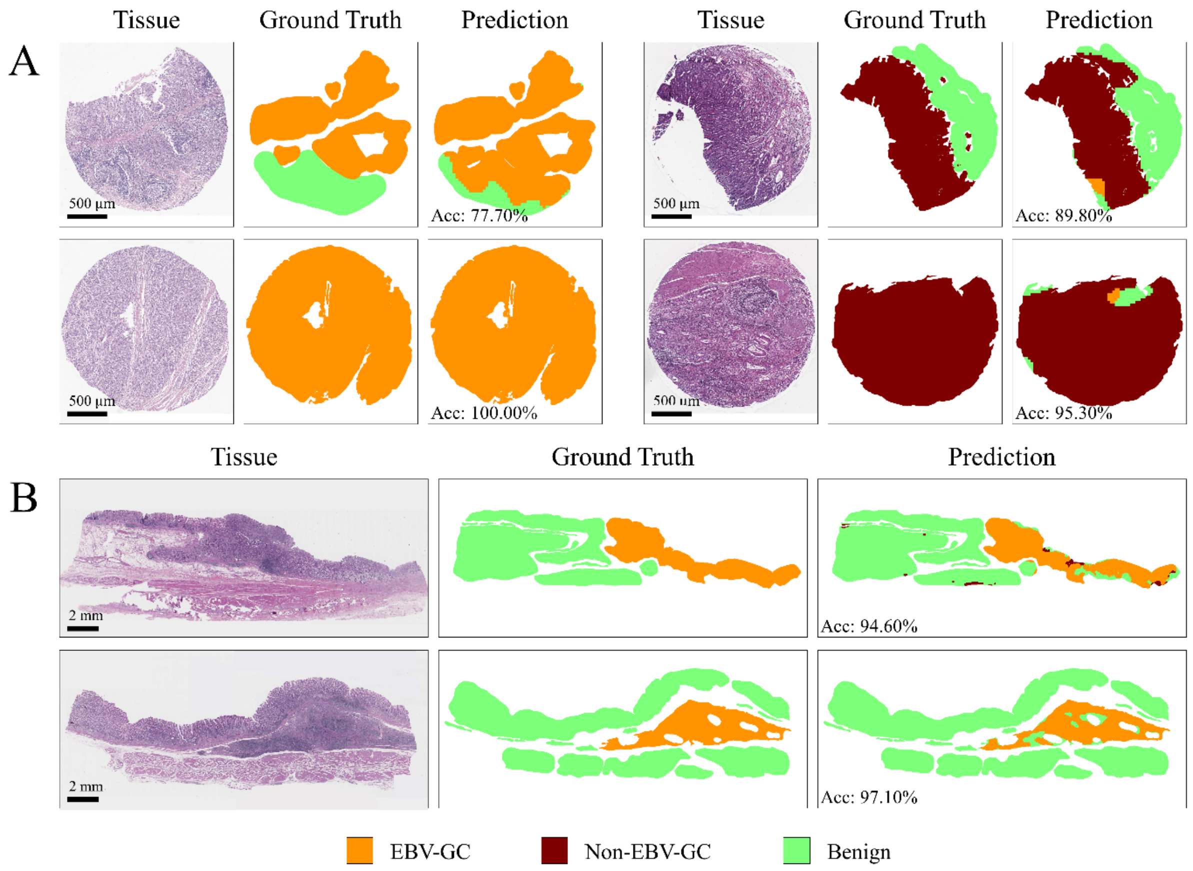

(A) TMAs and (B) WSIs. When comparing the ground truth and prediction results, the two maps agree well with both TMAs and WSIs.

© 2022 Vuong TTL et al. *JAMA Network Open.*

eFigure 3. Representative Prediction Maps of Biopsy Specimens

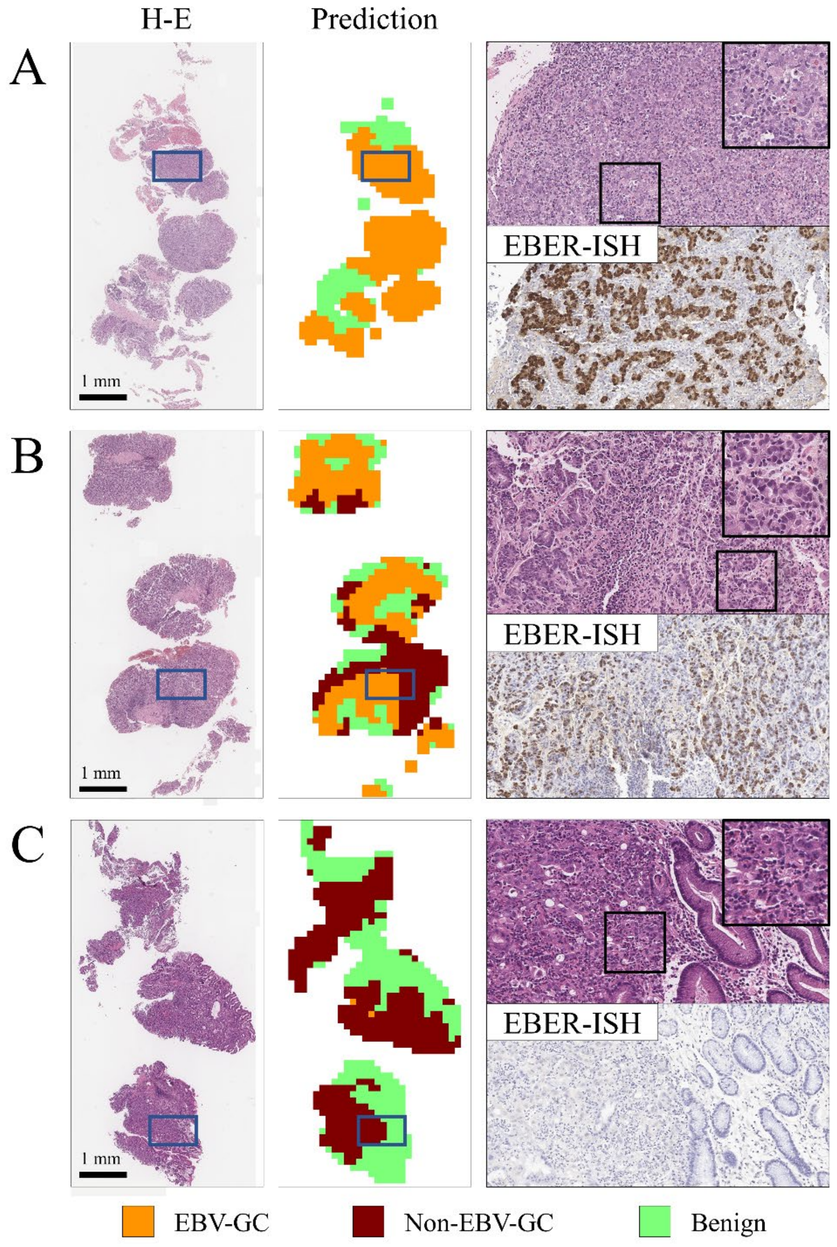

(A) A typical EBV-GC case with prominent lymphoid stroma and lace-like pattern is marked as EBV-GC in the prediction result. (B) Another EBV-GC case showing heterogeneous prediction results with no significant histologic differences between areas marked as EBV-GC and non-EBV-GC in the prediction map, although tumor cells were diffusely positive in EBER-ISH. (C) A case of non-EBV-GC with many infiltrating neutrophils was correctly classified as non-EBV-GC in the prediction map.

© 2022 Vuong TTL et al. *JAMA Network Open.*

eFigure 4. ROC Curves for EBV-GC Prediction in Biopsy Specimens

A

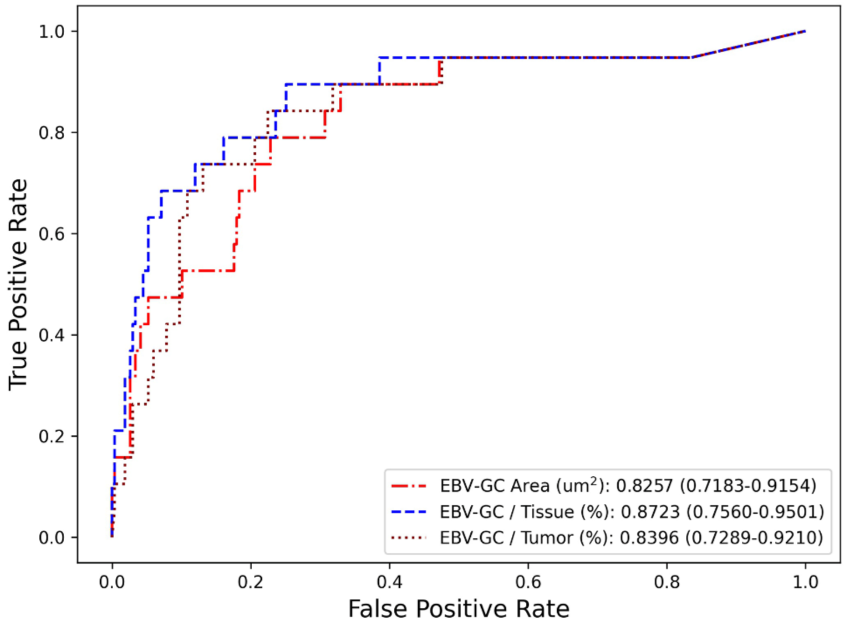

B

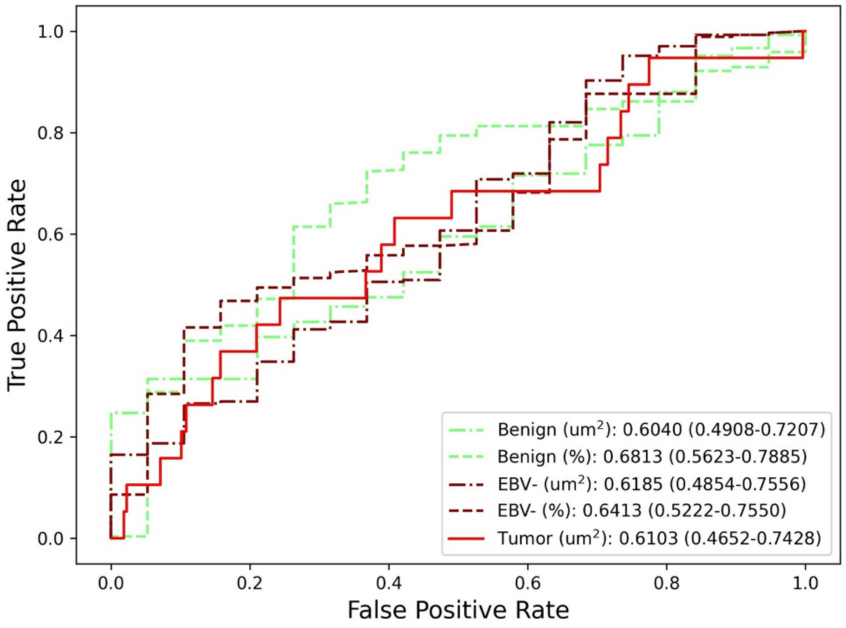

(A) EBV-GC Area, EBV-GC/Tissue (%), and EBV-GC/Tumor (%) showed superior AUC values and significant *p*-values (less than 0.001). Among them, EBV-GC/Tissue (%) was best for predicting EBV status. (B) Other measurements were not successful in predicting EBV status.

© 2022 Vuong TTL et al. *JAMA Network Open.*

eReferences.

1. He K, Zhang X, Ren S, Sun J. Deep residual learning for image recognition. 2016:770-778.
2. Sandler M, Howard A, Zhu M, Zhmoginov A, Chen L-C. Mobilenetv2: Inverted residuals and linear bottlenecks. presented at: 2018 IEEE/CVF Conference on Computer Vision and Pattern Recognition; 2018; Salt Lake City, UT, USA.
3. Tan M, Le Q. Efficientnet: Rethinking model scaling for convolutional neural networks. presented at: Proceedings of the 36th International Conference on Machine Learning; 2019; Long Beach, California.
4. Touvron H, Cord M, Douze M, Massa F, Sablayrolles A, Jégou H. Training data-efficient image transformers & distillation through attention. PMLR; 2021:10347-10357.
5. Hu J, Shen L, Sun G. Squeeze-and-excitation networks. presented at: 2018 IEEE/CVF Conference on Computer Vision and Pattern Recognition; 2018; Salt Lake City, UT, USA.
6. Ramachandran P, Zoph B, Le QV. Swish: a self-gated activation function. *arXiv preprint arXiv:171005941*. 2017;7(1):5. doi:10.48550/arXiv.1710.05941
7. Dosovitskiy A, Beyer L, Kolesnikov A, et al. An image is worth 16x16 words: Transformers for image recognition at scale. *arXiv preprint arXiv:201011929*. 2020;
8. Loshchilov I, Hutter F. Sgdr: Stochastic gradient descent with warm restarts. presented at: 5th International Conference on Learning Representations; 2017;
9. He K, Zhang X, Ren S, Sun J. Delving deep into rectifiers: Surpassing human-level performance on imagenet classification. 2015:1026-1034.

© 2022 Vuong TTL et al. *JAMA Network Open.*
